# Antioxidant Profile of Pepper (*Capsicum annuum* L.) Fruits Containing Diverse Levels of Capsaicinoids

**DOI:** 10.3390/antiox9090878

**Published:** 2020-09-17

**Authors:** José M. Palma, Fátima Terán, Alba Contreras-Ruiz, Marta Rodríguez-Ruiz, Francisco J. Corpas

**Affiliations:** 1 Group of Antioxidant, Free Radical and Nitric Oxide in Biotechnology, Food and Agriculture, Department Biochemistry, Cell and Molecular Biology of Plants, Estación Experimental del Zaidín, CSIC, 18008 Granada, Spain; fatimateca26@gmail.com (F.T.); acontreras@iata.csic.es (A.C.-R.); javier.corpas@eez.csic.es (F.J.C.); 2 Department Agricultural and Environmental Sciences, Universitat Jaume I, 12071 Castelló de la Plana, Spain; 3 Instituto de Agroquímica y Tecnología de Alimentos, IATA-CSIC, 46980 Paterna, Valencia, Spain; 4 Laboratório de Fisiologia do Desenvolvimiento Vegetal, Instituto de Biociências, Universidad de São Paulo, Cidade Universitária, São Paulo 05508-900, SP, Brazil; martarodriguezruiz@usp.br

**Keywords:** ascorbate, ascorbate–glutathione cycle, capsaicin, catalase, dihydrocapsaicin, glutathione, NADP-dehydrogenases, superoxide dismutase

## Abstract

*Capsicum* is the genus where a number of species and varieties have pungent features due to the exclusive content of capsaicinoids such as capsaicin and dihydrocapsaicin. In this work, the main enzymatic and non-enzymatic systems in pepper fruits from four varieties with different pungent capacity have been investigated at two ripening stages. Thus, a sweet pepper variety (Melchor) from California-type fruits and three autochthonous Spanish varieties which have different pungency levels were used, including Piquillo, Padrón and Alegría riojana. The capsaicinoids contents were determined in the pericarp and placenta from fruits, showing that these phenyl-propanoids were mainly localized in placenta. The activity profiles of catalase, total and isoenzymatic superoxide dismutase (SOD), the enzymes of the ascorbate–glutathione cycle (AGC) and four NADP-dehydrogenases indicate that some interaction with capsaicinoid metabolism seems to occur. Among the results obtained on enzymatic antioxidants, the role of Fe-SOD and the glutathione reductase from the AGC is highlighted. Additionally, it was found that ascorbate and glutathione contents were higher in those pepper fruits which displayed the greater contents of capsaicinoids. Taken together, all these data indicate that antioxidants may contribute to preserve capsaicinoids metabolism to maintain their functionality in a framework where NADPH is perhaps playing an essential role.

## 1. Introduction

Pepper (*Capsicum annuum* L.) fruits are one of the most consumed vegetables worldwide. Pepper fruits are mainly characterized by their high vitamin C and A and mineral contents [1,2,3,4,5,6,7,8]. Thus, about 60–80 g intake of fruits per day can provide 100 and 25% of recommended daily amounts of vitamin C and A, respectively [5,9]. Besides, this horticultural product contains important levels of other health-promoting substances with antioxidant capacity, and they include carotenoids, flavonoids and other polyphenols, among others [1,10,11,12].

The diversity of pepper varieties is quite high and they are basically differentiated by shape, size, pulp (pericarp) thickness and final color at the ripe stages. This diversity is also mirrored by the number of common names to designate pepper fruits which, in most cases, are used very locally. From culinary and gastronomic points of view, pepper fruits are mainly classified as sweet and hot depending on the absence or presence of capsaicin, respectively [4,5,12,13]. Within the sweet pepper (also amply known as bell pepper) varieties, three main types are distinguished according to their shape and size: California, Lamuyo and Dulce italiano. Hot peppers include the highest number of varieties and names including chili, habanero, jalapeño, paprika, chipotle and the Spanish Alegría riojana, Padrón and Piquillo used in this work, among others.

Capsaicin is exclusive to the genus *Capsicum* and is responsible for the pungency trait. According to the pungent level, pepper fruits are ranked on the so-called Scoville scale which assigns a score to each fruit variety. In this scale, the highest value for the most pungent pepper fruit variety is around 3 × 10^6^, pure capsaicin being 16 × 10^6^ [14,15,16,17,18]. Capsaicin is an alkaloid with a phenyl-propanoid nature which has given rise to a family of capsaicinoids composed of at least 22 primary compounds. Out of them, capsaicin and dihydrocapsaicin contribute to about 90–95% of total capsaicinoids present in most hot pepper varieties [19,20]. These compounds are mainly localized in the epidermal vacuoles of the placenta and the septum from fruits, and they can be separated and identified through the use of high-performance liquid chromatography associated with electrospray ionization mass spectrometry (HPLC-ESI/MS) [19,21,22]. Capsaicin is useful for pepper plants to avoid biting by insects and other animals since this chemical has repellent/insecticide capacity [23,24,25,26,27]. From a pharmacological perspective, the research carried out so far has shown that capsaicinoids, particularly capsaicin, have a diversity of biological and physiological functions in vitro, so they play roles as antioxidants, stimulants of the energetic metabolism, fat accumulating suppressors, anti-inflammatories, neurostimulants and apoptosis-alleviating agents in neurodegenerative disorders [20,28,29,30]. Regarding the mechanism of action, capsaicinoids act on a family of ion channels known as transient receptor potential (TRP) channels which, in mammals, are framed within the subtype TRP Vanilloid (TRPV1) [31,32]. It has been also found that in many types of cancers, the proapoptotic activity of capsaicin is also mediated by this TRPV, and the activation of the p53 tumor suppressing protein by a phosphorylation process is induced by capsaicin [33,34].

Another relevant feature of pepper fruits is the ripening process, visibly characterized by a shift in the fruit color from green to red, yellow, orange or purple depending on the variety. This event implies chlorophyll breakdown and synthesis of new carotenoids and anthocyanins, emission of organic volatiles, new protein synthesis and cleavage of existing ones and cell wall softening, among others [5,7,35,36,37,38,39]. Relevant differences between the transcriptomes from green immature and ripe pepper fruits have been also reported, involving thousands of genes [8,40] and references therein. From a redox viewpoint, it has been found that reactive oxygen species (ROS) metabolism is also affected during fruit ripening, leading to major changes in total soluble reducing equivalents and the antioxidant capacity in fruits [41]. The profile of the major non-enzymatic antioxidants, including ascorbate, glutathione, carotenoids and polyphenols, has been followed during ripening in pepper fruits [4,11,12,42,43,44,45,46], but less is known on how enzymatic antioxidants evolve with this physiological process. These enzyme systems basically include superoxide dismutase (SOD), catalase (CAT) and the ascorbate–glutathione cycle as the primary defense barriers against ROS, and some NADP-dehydrogenases as a secondary system to help the antioxidative enzymatic block. The profile of these enzymes throughout fruit ripening has been mostly carried out in sweet pepper [4,11,45,46], but scarce references have been reported on how those antioxidant enzymatic systems behave in the ripening of hot varieties [47,48,49]. Accordingly, using pepper varieties containing increasing capsaicin and dihydrocapsaicin contents, this work was aimed at characterizing the profile of the main antioxidants and their potential interaction with capsaicinoids during fruit ripening. This could provide a biochemical support and an added value for the particular features of each Spanish autochthonous cultivar that is included in the European Register of protected designations of origin for these horticultural products.

## 2. Materials and Methods

### 2.1. Plant Material

**Figure 1:**
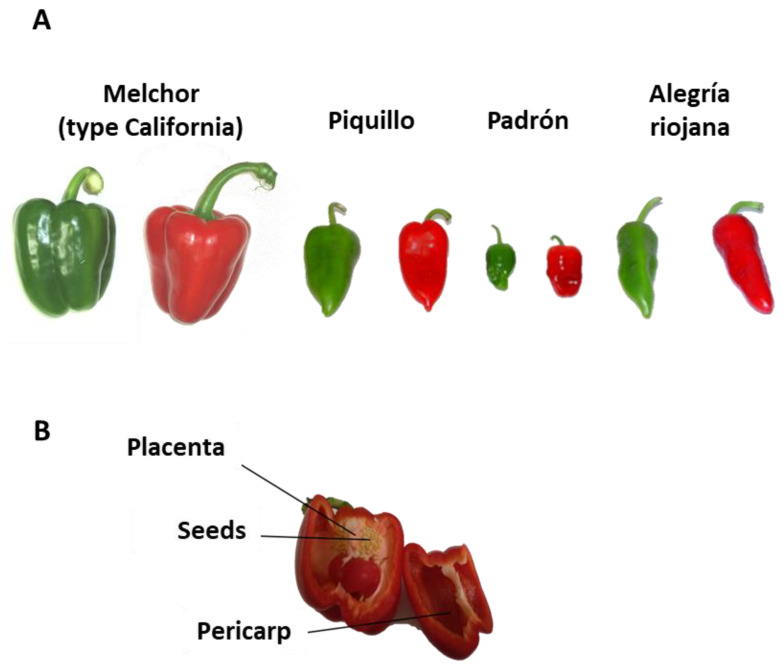
Representative pictures of plant materials used in this work. (**A**) Fruits from the four varieties at two ripening stages: green and ripe red. Melchor is a variety of California-type sweet pepper fruit. Piquillo Padrón and Alegría riojana contain different levels of capsaicin with the sequence Piquillo <<< Padrón < Alegría riojana. (**B**) Different parts of the pepper fruit.

Fruits from four pepper (*Capsicum annuum* L.) varieties were used in this work: California-type (sweet), obtained from plants grown in plastic-covered greenhouses (Zeraim Iberica/Syngenta Seeds, Ltd., El Ejido, Almería, Spain); Padrón (mild hot), provided by the Regulatory Council of Denomination of Origin “Pemento de Herbón” (Herbón, Coruña, Spain) and Piquillo (slightly hot) and Alegría riojana (quite hot), both provided by the Regulatory Council of Denomination of Origin “Pimiento del Piquillo-Lodosa” (Navarra, Spain). Padrón, Piquillo and Alegría riojana (onwards Alegría) fruits were obtained from plants grown in orchards under the local conditions. In all varieties, fruits at both green and red ripe stages were analyzed. Both stages were set according to marketing and consuming preferences as indicated by the growers. Green fruits did not show any ripening symptoms, mainly color shift, and they were totally green. Red fruits were harvested after several days they underwent the color change *in planta*. In all cases, fruits did not display any apparent damages. Figure 1A shows representative pictures of the different varieties used in this work, and in Table 1, comparative data on the mean fresh weight (g) of each type of fruit are given. Fresh weight data were obtained from 10 fruits of each variety at both maturation stages. After harvesting, in all fruits set for analyses, the pericarp and placenta (once seeds were discarded) were separated (Figure 1B), and each one was cut into small cubes (approximately 3–5 mm/side), frozen under liquid nitrogen and then stored at −80 °C until use. All biochemical parameters were determined thrice from 5 fruits in each variety and at the two ripening stages. As a whole, each mean was obtained from 15 assays.

### 2.2. Determination of Capsaicin and Dihydrocapsaicin by High-Performance Liquid Chromatography-Electrospray Mass Spectrometry (HPLC-ES/MS)

Samples were ground into a powder under liquid N_2_ and using an IKA^®^ A11 Basic mill (IKA Laboratories Inc., Tirat Carmel, Israel). For each sample, three extractions were made as follows. Plant materials (0.5 g powder) were suspended into 2.0 mL acetonitrile (AcN) containing 100 ppm N-[(3,4-dimethoxyphenyl)methyl]-4-methyl-octanamide (DMBMO), as an internal standard. Mixtures were incubated in the following sequence: 1 h at room temperature and darkness with continuous shaking; 65 °C and darkness for 1 h and short shakings every 15 min; and 1 h at room temperature in the dark. Then, samples were centrifuged at 16,000× *g* and room temperature for 15 min. Supernatants were passed through 0.22 μm pore size polyvinylidene fluoride filters and were used for analysis through HPLC-ESI/MS with mode multiple reaction monitoring (MRM). An XBridge 2.1 × 10 mm pre-column and an XBridge 2.1 × 100 mm C18 3.5 µm column (Waters Corporation, Milford, MA, USA) were used connected to an Allience 2695 HPLC system coupled to a Micromass Quattro micro API triple quadrupole mass spectrometer both obtained from the Waters Corporation. The chromatography was run at a flux of 0.3 mL/min with temperatures of 35 °C for the column and 5 °C for the auto-injector, with 5 μL being injected per sample. The gradient used was: 6 min with AcN:H_2_O (60:40) containing 0.1% (*v/v*) formic acid; 10 + 5 min with AcN:H_2_O (90:10); and 20 + 4 min with AcN:H_2_O (60:40). Standard curves were prepared using pure capsaicin and dihydrocapsaicin (Cayman Chemical, Ann Arbor, MI, USA). Under these conditions, the retention time for capsaicin and dihydrocapsaicin was 1.88 and 2.24 min, respectively. The concentration of capsaicinoids was expressed as μg g^−1^ of fresh weight (FW).

### 2.3. Detection and Quantification of Ascorbate, GSH and GSSG by High-Performance Liquid Chromatography-Electrospray Mass Spectrometry (LC-ES/MS)

Pericarps and placentas were ground under liquid N_2_ with a pestle and a mortar. Then, 0.4 g of powdered tissues was suspended into 1 mL of 0.1 M HCl and spun for 20 min at 15,000× *g* and 4 °C. Supernatants were passed through polyvinylidene fluoride filters (0.22-μm pore size) and analyzed immediately. All procedures were performed at 4 °C with protection from light to prevent potential degradation of the metabolites. Samples were analyzed by liquid chromatography–electrospray/mass spectrometry (LC-ES/MS) using the HPLC system and mass spectrometer indicated above. HPLC runs were performed with an Atlantis^®^ T3 3 μm 2.1 × 100 mm column (Waters Corporation). The MassLynx 4.1 software package (Waters Corporation) was used for the instrument control and collection, analysis and management of data. With this method, the simultaneous detection and quantification of ascorbate, reduced (GSH), and oxidized (GSSG) glutathione is achieved [7,50]. The analytes concentration was calculated with the use of external standards and expressed with reference to fresh weight (FW).

### 2.4. Preparation of Crude Extracts for Enzyme Activity

Protein extracts from pericarps and placentas were powdered under liquid nitrogen and then suspended in 0.1 M Tris-HCl buffer, pH 8.0, containing 1 mM EDTA, 0.1% (*v/v*) Triton X-100 and 10% (*v/v*) glycerol, in a final 1:1 (*w:v*) plant material/buffer ratio. Crude extracts were centrifuged at 15,000× *g* for 30 min and the supernatants were used for enzymatic assays.

### 2.5. Enzyme Activity Assays

All enzyme activities were determined using an Evolution 201 UV–visible spectrophotometer (Thermo Fisher Scientific, Waltham, MA, USA). Catalase (EC 1.11.1.6) activity was determined by following the H_2_O_2_ breakdown at 240 nm [51]. Ascorbate peroxidase (APX; EC 1.11.1.11) was monitored at 290 nm by plotting the initial ascorbate oxidation by H_2_O_2_ [52]. Monodehydroascorbate reductase (MDAR; EC 1.6.5.4) activity was assayed by following the monodehydroascorbate-dependent NADH oxidation. In these assays, monodehydroascorbate was generated through the ascorbate/ascorbate oxidase system as reported earlier [53]. The monodehydroascorbate-independent NADH oxidation rate (without ascorbate oxidase and ascorbate) was deducted from the monodehydroascorbate-dependent reaction. Dehydroascorbate reductase (DHAR, EC 1.8.5.1) activity was measured by monitoring at 265 nm the increase in ascorbate formation, with the use of a N_2_-saturated buffer. The reaction rate was corrected by the non-enzymatic dehydroascorbate reduction through reduced glutathione (GSH). A 0.98 factor was also considered, due to the little contribution to the absorbance by oxidized glutathione (GSSG) [54]. Glutathione reductase (GR; EC 1.6.4.2) activity was analyzed by following at 340 nm the NADPH oxidation associated with the reduction of GSSG to GSH [55]. The GR reaction rate was corrected for the very small, non-enzymatic NADPH oxidation by GSSG.

Total SOD (EC 1.15.1.1) activity was determined by the ferricytochrome *c* reduction method, with the system xanthine/xanthine oxidase as a superoxide radical (O_2_^·−^) source. One activity unit was defined as the amount of protein necessary to inhibit 50% of the cytochrome *c* reduction [56]. For the analysis of the SOD isoenzyme profile, proteins from crude extracts were separated by vertical non-denaturing PAGEs on 10% acrylamide gels, using a Mini-Protean III Tetra Cell system (Bio-Rad Laboratories, Hercules, CA, USA). SOD isozymes were detected in the gels as acromatic bands over a purple background by a specific staining based in the photochemical reduction method of nitroblue tetrazolium (NBT) [57]. For the identification of the different SOD isozymes, before the staining procedure, pre-incubation of gels was carried out in the presence of specific inhibitors, either 5 mM KCN or 5 mM H_2_O_2_. Copper- and zinc-containing SODs (CuZn-SODs) are inhibited by both KCN and H_2_O_2_; iron-containing SODs (Fe-SODs) are inactivated by H_2_O_2_; and Mn-SODs are resistant to both inhibitors [58,59].

NADP-dependent dehydrogenase (NADP-DHs) activities were determined by recording the NADPH formation at 340 nm and 25 °C. The assay was performed in a reaction medium containing 50 mM HEPES [(4-(2-hydroxyethyl)-1-piperazineethanesulfonic acid)], pH 7.6, 2 mM MgCl_2_ and 0.8 mM NADP. Each enzymatic reaction was initiated by the addition of the respective specific substrates [46]. For the glucose-6-phosphate dehydrogenase (G6PDH, EC 1.1.1.49) activity, the reaction started after the addition of 5 mM glucose-6-phosphate. To monitor 6-phosphogluconate dehydrogenase (6PGDH, EC 1.1.1.44) activity, 5 mM 6-phosphogluconate was used as the substrate. NADP-isocitrate dehydrogenase (NADP-ICDH, EC 1.1.1.42) activity was triggered with 10 mM 2R,3S-isocitrate [60,61]. For the NADP-malic enzyme (NADP-ME, EC 1.1.1.40) activity, the reaction was initiated with 1 mM L-malate [62].

Protein concentration in samples was determined by the Bradford method [63], using the Bio-Rad protein assay solution (Bio-Rad Laboratories) and bovine serum albumin as the standard.

### 2.6. Immunoblot Analysis

Proteins separated by native-PAGE (10% acrylamide) and SDS-PAGE (12% acrylamide) were transferred onto polyvinylidene difluoride (PVDF) membranes, using a Trans-Blot SD equipment (Bio-Rad Laboratories). The transfer buffer used was 10 mM N-cyclohexyl-3-aminopropanesulfonic acid (CAPS), pH 11.0, 10% (*v/v*) methanol. Runs were developed at 1.5 mA/cm^2^ membrane for 2 h [64]. After the protein transfer, membranes were processed for further blotting assays. An antibody against Fe-SOD from pepper fruits (dilution 1:5000) was used. The antibody-recognizing proteins were visualized using the ClarityTM Western ECL Substrate kit (Thermo Fisher Scientific) following the manufacturer’s instructions.

### 2.7. Statistical Analysis

One-way ANOVA was used for the comparisons between means of capsaicin and dihydrocapsaicin contents using the Statgraphics Centurion program (Statgraphics Technologies, Inc., Madrid, Spain). For other parameters, the *t*-student test was used to detect differences between the two ripening stages of each variety. In both the ANOVA and *t*-student, values for *p* < 0.05 were considered different with statistical significance.

## 3. Results

In this work, pepper fruits from four varieties with different pungency tastes were investigated. Thus, the concentration of the main capsaicinoids, capsaicin and dihydrocapsaicin, was analyzed. As shown in Table 2, Melchor, which is a sweet variety, did not contain any of the capsaicinoids, and Piquillo only displayed little values both in green and red fruits, with placenta being the tissue where both metabolites were present in higher amounts. Regarding Padrón and Alegría, both varieties showed high capsaicinoid contents, with less amounts in the pericarp and the major levels being clearly observed in the placenta. In these two last varieties, the concentration of capsaicin and dihydrocapsaicin was remarkably increased in ripe red fruits.

**Figure 2:**
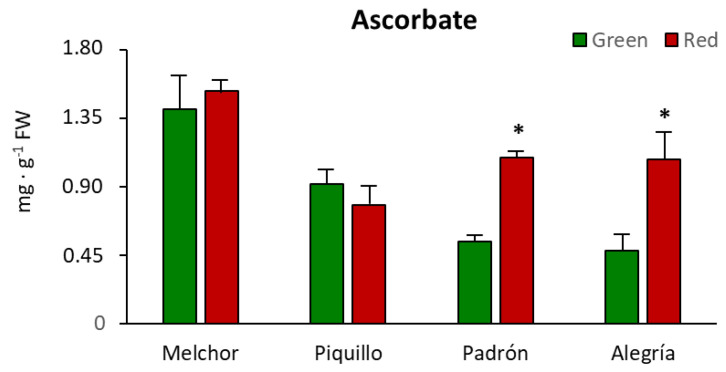
Ascorbate content in pericarp from fruits of four pepper varieties at two ripening stages. Data are the means ± SEM of three replicates determined from five fruits of the four varieties and at the two ripening stages. Asterisks indicate significant differences of red fruits with respect to green fruits for each variety (*t*-student, *p* < 0.05). FW, fresh weight.

**Figure 3:**
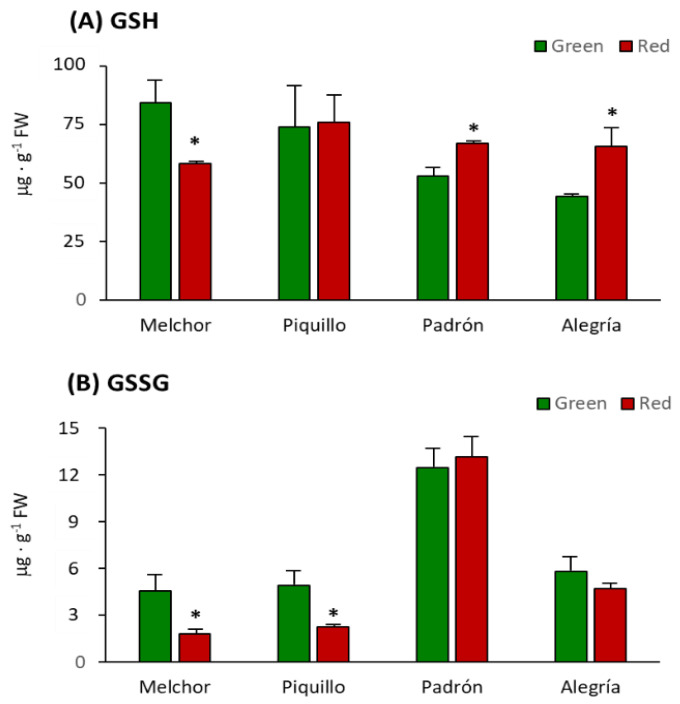
Reduced (GSH) and oxidized (GSSG) glutathione contents in pericarp from fruits of four pepper varieties at two ripening stages. (**A**) GSH. (**B**) GSSG. Data are the means ± SEM of three replicates determined from five fruits of the four varieties and at the two ripening stages. Asterisks indicate significant differences of red fruits with respect to green fruits for each variety (*t*-student, *p* < 0.05). FW, fresh weight.

As shown in Figure 2, the higher ascorbate concentration was found in Melchor, and this parameter only changed due to ripening in the two varieties with higher capsaicinoid levels, Padrón and Alegría. In both, ascorbate was significantly enhanced after fruits ripened. Likewise, this tendency also occurred with GSH, which only increased significantly in Padrón and Alegría after ripening, whereas it lowered in Melchor after this physiological process took place (Figure 3A). The oxidized form of glutathione (GSSG) diminished in Melchor and Piquillo ripened fruits and no changes were observed in Padrón and Alegría. As indicated in Table 3, total glutathione content (GSH + GSSG) increased in Padrón and Alegría and lowered in Melchor after ripening. The ratio GSH/GSSG was enhanced by ripening in the four varieties, thus indicating a shift to a higher reducing environment (Table 3).

**Figure 4:**
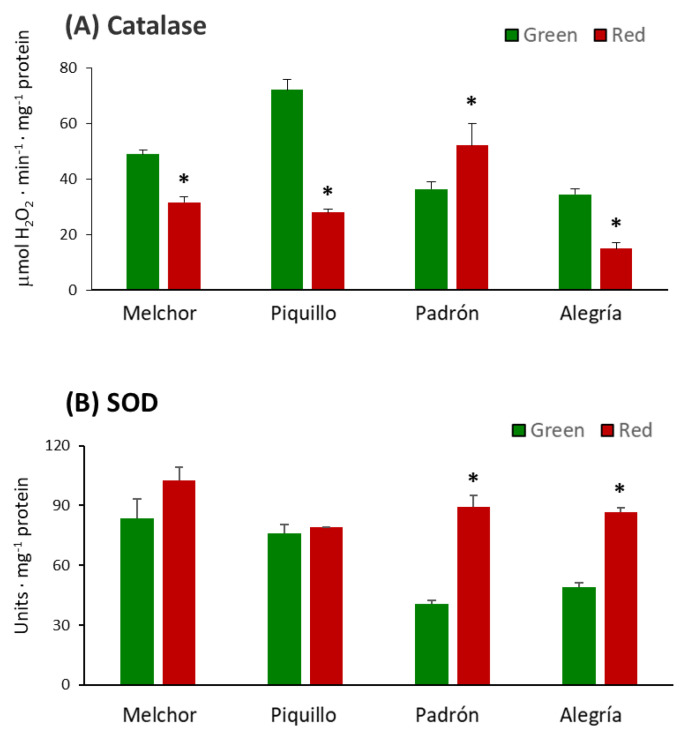
Catalase and superoxide dismutase (SOD) activity in pericarp from fruits of four pepper varieties at two ripening stages. (**A**), catalase. (**B**), SOD. Data are the means ± SEM of three replicates determined from five fruits of the four varieties and at the two ripening stages. Asterisks indicate significant differences of red fruits with respect to green fruits for each variety (*t*-student, *p* < 0.05).

**Figure 5:**
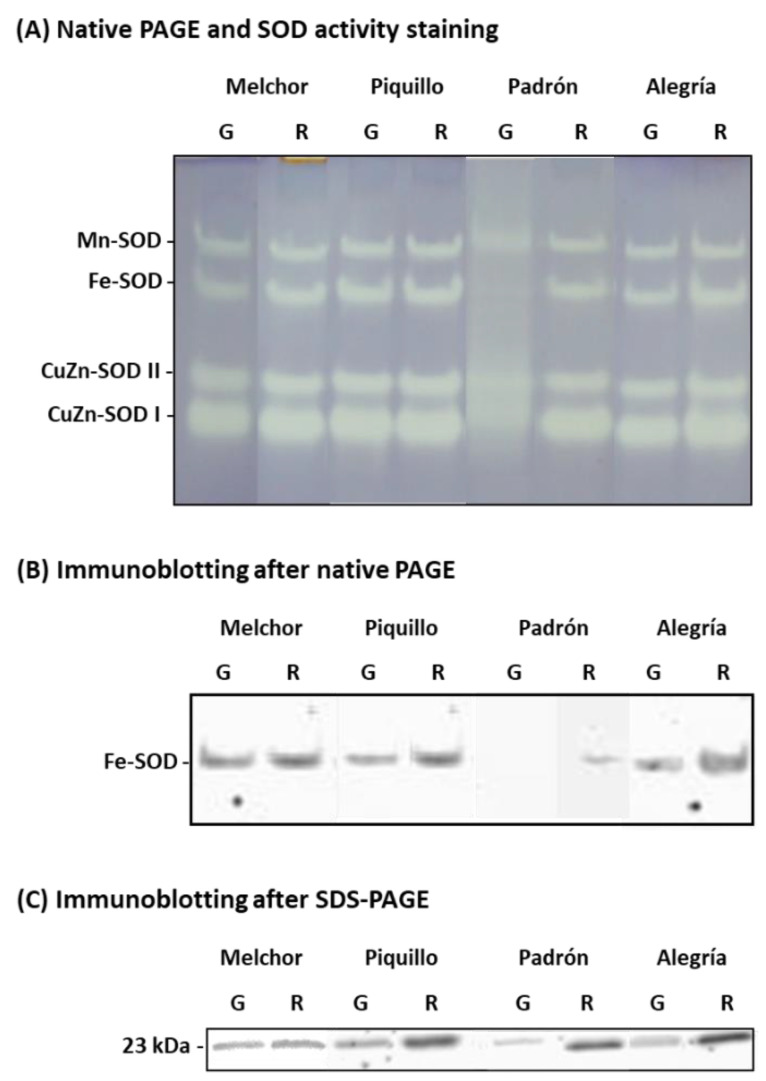
Isoenzymatic superoxide dismutase (SOD) pattern in pericarp from fruits of four pepper varieties at two ripening stages. (**A**) Native PAGE on 10% acrylamide gels and further in-gel SOD activity staining by the NBT reduction method; 34 μg protein per well was loaded. (**B**) Immunoblotting after native PAGE on 10% acrylamide gels. (**C**) Immunoblotting after SDS-PAGE on 12% acrylamide gels. In both immunoblotting assays, an antibody against Fe-SOD from pepper fruits (dilution 1:5000) was used. In panel B, the mobility of the detected bands was similar to the one observed for Fe-SOD in panel A. The monomeric molecular size of the cross-reacting bands (23 kDa) is indicated on the left in panel C. Data are representative of at least three independent experiments where different samples from each variety and ripening stage were used. G, green fruits. R, red fruits.

The activity of the main enzymatic antioxidants was studied. Catalase was significantly lower in ripe fruits from all varieties except for Padrón, where the activity increased after ripening (Figure 4A). SOD activity increased as a consequence of ripening but only significantly in Padrón and Alegría. No changes were observed in the Piquillo variety at the two stages (Figure 4B). This SOD activity pattern was partially confirmed by the analysis of the isoenzymatic profile. Thus, in the Padrón variety, no Fe-SOD activity was detected in green fruits, whereas this isozyme appeared in red fruits (Figure 5A). Additionally, CuZn-SOD I and II were also higher in ripe fruits than in green ones. Regarding the Alegría variety, it was observed that Fe-SOD and CuZn-SOD II were more prominent in red fruits than in green fruits (Figure 5A). To seek for the possible reason of the absence of Fe-SOD activity in the Padrón variety, immunoblot assays were performed under native and denaturing conditions. Thus, after native PAGE and Western blotting analysis using an antibody against an Fe-SOD from pepper fruits, no cross-reacting bands were observed in green fruits from the Padrón variety. Additionally, the use of this approach confirmed that the activity pattern observed in Alegría was due to a higher Fe-SOD protein amount in red fruits (Figure 5B). To further check that Padrón did not contain the Fe-SOD protein, SDS-PAGE and Western blotting was achieved. In all samples, a cross-reacting band, characteristic of the plant Fe-SOD monomeric size (23 kDa), was detected, including green fruits from the Padrón variety, although with a very low quantity (Figure 5C). This indicates that this isozyme is present in this variety, but in such a little amount that its contribution to the total SOD activity is possibly irrelevant.

**Figure 6:**
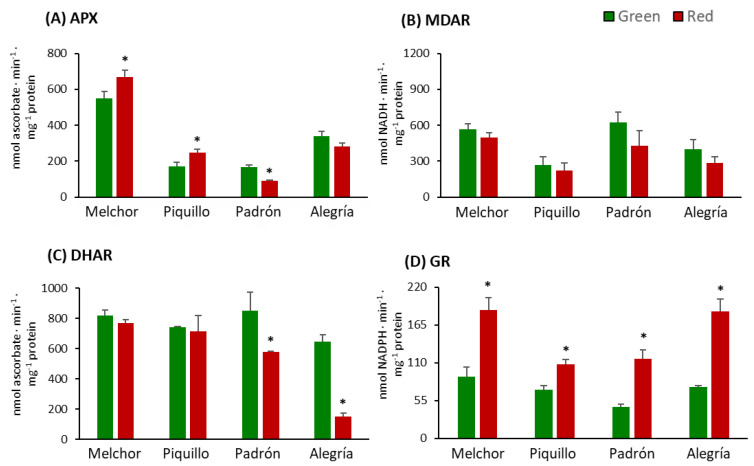
Activity of enzymes from the ascorbate–-glutathione cycle in pericarp from fruits of four pepper varieties at two ripening stages. (**A**) Ascorbate peroxidase (APX). (**B**) Monodehydroascorbate reductase (MDAR). (**C**) Dehydroascorbate reductase (DHAR). (**D**) Glutathione reductase (GR). Data are the means ± SEM of three replicates determined from five fruits of the four varieties and at the two ripening stages. Asterisks indicate significant differences of red fruits with respect to green fruits for each variety (*t*-student, *p* < 0.05).

The enzymatic side of the AGC was analyzed, following the activity of APX, MDAR, DHAR and GR. APX was little, but significantly enhanced in ripe fruits with respect to green fruits in Melchor and Piquillo, and lower in red fruits from Padrón (Figure 6A). Regarding MDAR, this enzyme did not show significant changes upon ripening in the four varieties (Figure 6B). DHAR was only significantly lower in red fruits from those varieties with a high capsaicinoid content, Padrón and Alegría (Figure 6C). Finally, all varieties displayed significant enhanced GR activity after ripening (Figure 6D).

**Figure 7:**
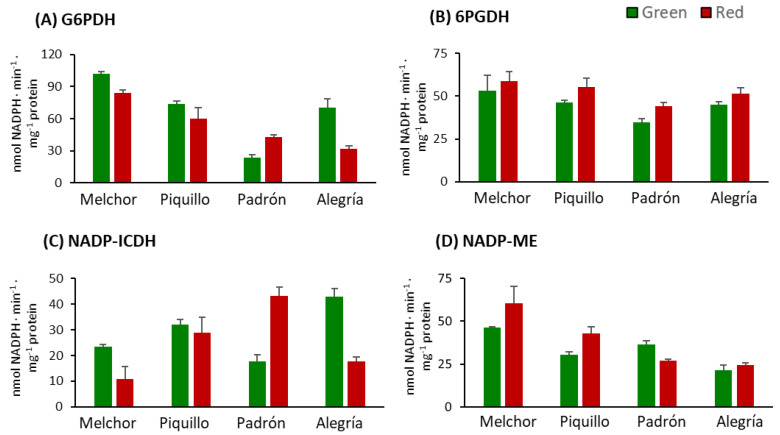
Activity of NADP-dehydrogenases in pericarp from fruits of four pepper varieties at two ripening stages. (**A**) Glucose-6-phosphate dehydrogenase (G6PDH). (**B**) 6-Phosphogluconate dehydrogenase (6PGDH). (**C**) NADP-dependent isocitrate dehydrogenase (ICDH). (**D**) NADP-dependent malic enzyme (ME). Data are the means ± SEM of three replicates determined from five fruits of the four varieties and at the two ripening stages.

Regarding the activity profile of NADP-dependent dehydrogenases (NADP-DHs), four eznymes were studied: G6PDH, 6PGDH, NADP-ICDH and NADP-ME. G6PDH and NADP-ICDH displayed parallel profiles with lower activities in red than in green fruits in the varieties Melchor and Alegría, but enhanced activity after fruits from the Padrón variety ripened (Figure 7A,C). No changes in those enzymatic systems were observed in fruits from the Piquillo variety. With respect to 6PGDH, this activity only changed in Padrón, with enhancement after ripening (Figure 7B). NADP-ME showed disparate evolution depending on the varieties. Thus, it increased in Melchor and Piquillo upon ripening and lowered in Padrón, with no changes in Alegría (Figure 7D).

## 4. Discussion

### 4.1. The Experimental Design Provided a Gradual Capsaicin Concentration Depending on the Pepper Variety and the Ripening Stage

Pepper varieties used in this work were selected because of their different pungency levels according to consumers taste, which is the basis where the Scoville scale resides. All four varieties are common in Spanish food markets and their culinary uses are diverse. Melchor is a type of sweet pepper characterized by its consistency and appropriateness for different purposes. This variety, along with other sweet pepper varieties, provides the high production figures in Spain. Its tasting features in either green or red frame this variety in the non-pungent fruits’ group. Piquillo is an autochthonous variety from northern Spain and its main phenotypic feature is its triangle shape with a sharp-peaked extreme. Upon intake, it is characterized by a very slight pleasant pungency, but it is only consumed in its ripe red stage. Padrón is characteristic of and originally from northwestern Spain, although lately it is also cultivated in many other lands in the Mediterranean area. These fruits are small, and they are usually consumed as green after cooking. Commonly, in the green stage, they show a very slight spicy taste, but it is in the red stage that it is not consumed due to its strong pungency. Finally, Alegría riojana (which might be translated as Riojan joy) is also autochthonous from northern Spain and it is usually used as spice in the red stage. Both green and red, but mainly red, fruits from this variety are extremely pungent.

With this tasting background and considering the antioxidant quality attributed to capsaicinoids [20,28,29], we aimed, in this work, to investigate the potential influence of these compounds (capsaicin plus dihydrocapsaicin; Cap+DiCap) in the profile of the main enzymatic and non-enzymatic antioxidants of pepper fruits containing different levels of these alkaloids. Our experimental design established a gradual scale from null values of Cap+DiCap, both in green and red ripe stages (Melchor), to red Alegría which contained high levels of the two capsaicinoids. The content of the Cap+DiCap couple matched with the tasting scale and the higher values, as expected, were found in the placenta in the three pungent varieties. Based on these data, we found quite the appropriate selection of these varieties and ripening stages to target our objective.

Except for Alegría which has been scarcely used for research purposes so far, reports on the other three varieties can be found in the literature. Thus, the Melchor variety has been used to decipher the mechanisms involved in fruit ripening [18,38,65,66], where some of their antioxidant systems have been reported to be involved [67]. The Piquillo variety was used as a model to address the effects triggered by infection with *Verticillum* [68,69,70,71] and how to protect pepper plants against it through diverse practices [72,73], as well as to investigate the effect of sanitized sewage sludge on the growth, yield, fruit quality, soil microbial community and physiology of pepper plants [74,75]. On the other hand, the Padrón variety was set, for example, to investigate either how wounding induces local resistance but systemic susceptibility to *Botrytis cinerea* in pepper plants [76], as a reference to assess real-time PCR as a method for determining the presence of *Verticillium dahliae* in distinct solanaceae species [77], or to study the virulence and pathogenesis issues of *Phytophthora capsici* [78], among others.

### 4.2. The Ripening Stage and the Capsaicinoids Content Alter the Metabolism of Enzymatic Antioxidants

The profile of antioxidant enzymes during the ripening process has been investigated in pepper fruits previously but, to our knowledge, no comparisons have been made between varieties with different capsaicinoid contents. Thus, for example, in California-type pepper fruits, it has been reported that the catalase activity decreases as the fruit ripens [79,80] and this event is due to the post-translational modification (PTMs) underwent by the enzyme and promoted by ROS and reactive nitrogen species (RNS) derived from nitric oxide (NO) [41,81]. In fact, it has been proved that the ripening of pepper fruits is controlled by NO [8,40,80]. This inhibitory effect of ripening in the catalase activity also occurred in the same California-type fruits subjected to storage at 20 °C [80], in other sweet pepper varieties from Lamuyo and Dulce italiano types [4], and during the ripening of hot pepper Kulai [49]. Our data on the Melchor, Piquillo and Alegría varieties confirm this activity pattern of the catalase activity, although, interestingly, this profile is opposite in the Padrón variety where catalase activity increases in ripe fruits. This same increasing catalase activity was reported in hot pepper varieties either under saline stress conditions [47] or in preventing seed browning during low-temperature storage [48].

Regarding SOD, the total activity was higher in ripe fruits from those varieties which contained a higher capsaicinoids content, namely Padrón and Alegría. In Alegría, this higher activity seems basically to be due to an enhancement of the isozymes Fe-SOD and CuZn-SOD II, whereas in Padrón, the presence of Fe-SOD (nearly absent in green fruits) and the higher activity of both CuZn-SODs could be responsible for such changes. Due to this interesting behavior of the Fe-SOD isozyme in the Padrón variety, complementary immunoblot analyses were performed using an antibody against the isozyme from pepper fruits. Thus, by Western blotting after both non-denaturing- and SDS-PAGEs, it was confirmed that the negligible Fe-SOD activity in ripe Padrón fruits was due to the little amounts of its corresponding protein, whose monomer (23 kDa) could only be detected after SDS-PAGE. This issue needs to be further investigated at the molecular level (gene and protein expression) since it means that it might be an identity feature of this pepper variety. The SOD activity has been also studied earlier in pepper varieties including some of those included in the present work. So, recently, it has been reported that the SOD isoenzyme pattern and gene expression of California-type pepper fruits are regulated by ripening and NO [67], and this enzymatic system from sweet pepper is also involved in the response against low temperatures [4] and the storage of fruits at 20 °C [79], as well as in the “accommodation” of fruits to nitrogen deprivation during plant growth [82]. The isoenzymatic SOD pattern was also investigated in the plastid population from sweet pepper fruits of different California-type varieties, and a protective role of these organelles by the different SOD internal isozymes during ripening was reported [45]. In the Piquillo variety, it was found that SOD is involved in the association of pepper plants with arbuscular mycorrhizal fungi (AMF) to avoid the negative effects promoted by *Verticillum* [73]. A number of studies have reported the involvement of SOD from hot pepper in diverse processes including ripening and post-harvest [49,83], salt stress [47,84], storage at low temperature [48] and iodine bio-fortification practices to improve fruit quality [85].

The activity of the four enzymes of the ascorbate–glutathione cycle (AGC), APX, MDAR, DHAR and GR, were analyzed in this work. APX is responsible for the direct scavenging of hydrogen peroxide (H_2_O_2_) using ascorbate as the electron donor, whereas MDAR and DHAR restore the reduced status of ascorbate using NAD(P)H and GSH, respectively. The last step of the AGC is carried out by GR, an enzyme which converts the oxidized form of glutathione (GSSG) to the reduced form (GSH) with the use of NADPH as the reducing power. In our experimental design, the most remarkable response of this cycle was found at the GR side which was significantly enhanced in ripe fruits from all four varieties. The profile of these AGC enzymes has been investigated in pepper fruits from diverse varieties, both sweet and hot, and different trends have been reported depending on the experimental conditions, including ripening, post-harvest, salt stress, defense mechanisms or bioremediation practices [4,11,45,49,75,82,83,86]. In our case, it is remarkable that APX behaved oppositely in sweet and hot varieties, with the activity increasing in ripe fruits from Melchor and Piquillo and decreasing in hot ripe fruits from Padrón and Alegría. MDAR and DHAR shared a similar trend with lower values in ripe fruits, but only significant in MDAR from Padrón and Alegría. According to the activity profile of APX, MDAR and DHAR from green to red stages, it could be hypothesized that the cycle seems to be operative in the first steps which involve direct ascorbate metabolism, but more research at different levels is necessary to obtain a whole picture of this antioxidant metabolic pathway. According to our results, it seems that hot peppers have less capacity to recycle ascorbate but all varieties showed a great potentiality to provide GSH.

The activity pattern displayed by the NADP-dehydrogenases (NADP-DHs) can be framed in three main features: (i) the behavior of the two varieties with less Cap+DiCap levels (Melchor and Piquillo) was quite similar with slight, although not strongly significant, decreases in G6PDH and little, although not significant enough, decreases in 6PGDH and NADP-ME in ripe fruits; (ii) except for NADP-ME, all other NADP-DHs rose after ripening in the Padrón variety (high capsaicinoids content), and this suggests a higher NADPH availability for different purposes in ripe fruits from this variety; and (iii) interestingly, the behavior of these enzymatic systems in the other variety with high capsaicinoids content was different to that showed by Padrón. Thus, green fruits from Alegría seemed to have higher capacity to generate NADPH. To our knowledge, no reports on NADP-DHs from hot pepper fruits have been published previously, and the only data concerning these NADP/NADPH systems in pepper refer to sweet varieties. Our data mostly confirm those previously found for other California-type pepper varieties [46]. Recent data report that pepper fruit NADP-DHs are not only influenced by ripening in the Melchor variety [8], but also by NO through diverse PTMs [87,88]. Moreover, it was also found that NADP-DHs are involved in the response of sweet pepper plants to stress exerted by high Cd levels [89].

### 4.3. The Higher Capsaicinoids Level the Higher Ascorbate and Glutathione Content

Capsaicinoids, specially capsaicin, have been reported to have, among others, antioxidant properties [20,28,29,30]. In pepper fruits containing these alkaloids, this feature is quite interesting since these horticultural products are one of those with the highest ascorbate levels [5], with ascorbate being perhaps the most paradigmatic molecular antioxidant for living beings. In fact, ascorbate is one of the parameters which is commonly determined in (sweet and hot) pepper fruit research including either ripening and post-harvest, any type of stress (biotic, abiotic and environmental) or culture practices [4,5,7,11,14,18,45,47,49,75,84,90]. As an appraisal of the potential roles attributed to ascorbate in pepper fruits, it was proposed that in the sweet varieties, ascorbate functions as a redox buffer to balance the great metabolic changes which undergo during ripening [5,7]. Regarding the hottest varieties (Padrón and Alegría), the pattern observed in this work, where ascorbate levels increased in those fruits, was also reported earlier for diverse hot pepper varieties [14,18,49,90]. Perhaps, the redox stabilizing role of ascorbate indicated above for sweet pepper could be also applicable to hot varieties to assure the capsaicinoids level. In fact, it was proved that during the capsaicinoids oxidation catalyzed by peroxidases, capsaicinoid radicals are formed, and ascorbate rapidly reduces capsaicinoid radicals, this being an important cue for capsaicinoid content and preservation in pepper fruits [91].

Glutathione is a ubiquitous and powerful antioxidant in eukaryotes [92]. In spite of its relevant role in many biological processes, this tripeptide has been less investigated in pepper fruits, mainly associated to ripening, or bioremediation purposes [49,75,82,93]. However, not much information is available on glutathione metabolism in capsaicinoids-containing pepper varieties. This work provides the first comparison of the levels of both GSH and GSSG in different pepper varieties containing gradual amounts of capsaicinoids. It is noteworthy that, whereas the total glutathione content (GSH + GSSG) did not change or decrease after ripening in the varieties with no or very few capsaicinoids (Merlchor and Piquillo), in the hot varieties, this parameter augmented in mature fruits. This was due to the evolution of the reduced form of GSH during those physiological processes. This higher content of GSH in ripe fruits found in the hottest pepper varieties could be due to an enhanced GR activity. In these cases, the enzyme GR is perhaps playing a role not linked to the AGC. GSH could be used, in cooperation with ascorbate, to preserve the capsaicinoids functionality in these hot varieties. However, more research is necessary to bring light to this emerging subject. Besides, GSH could be also driven to signaling processes by either glutathionylation events (another PTM), or as *S*-nitrosoglutathione, a chemical form which allows transporting NO among cells and tissues [50,94,95,96]. GR uses NADPH to achieve the reduction of glutathione. NADPH is also essential in intermediate steps of capsaicin biosynthesis [97]. These eventualities point towards the necessity of investigating the interaction capsaicinoids–ascorbate–glutathione–NADPH in more detail, especially after the perspective of considering NADPH as a quality footprint in horticultural crops, as it has been proposed recently [98].

## 5. Conclusions and Future Prospects

The obtained data in this work point towards a close relationship among capsaicinoids and the antioxidant systems in pepper fruits. This interaction seems to maintain a redox and functional homeostasis to preserve the role of capsaicinoids with the cooperation of antioxidants, basically ascorbate and glutathione. However, some antioxidant enzymatic systems are also involved. The exclusivity of capsaicinoid metabolism in *Capsicum* species makes this research more attractive to look for an exclusive model that could provide interesting information at the plant physiological level, but also considering the pharmacological and nutraceutical uses of hot pepper fruits, based mainly on the content of capsaicinoids but also on vitamins C and A. On the other hand, another interesting cue is opened. The role of Fe-SOD needs to be investigated in pepper fruit physiology due to the diverse behavior of this isozyme among varieties. Fe-SOD has been localized in peroxisomes from pepper fruits [5,99] and lately its gene expression profile has been reported in sweet pepper during ripening and under NO treatment. Overall, the interaction of NO in pungent pepper fruits is another issue that deserves to be investigated. Furthermore, this characterization contributes to providing a biochemical antioxidant pattern for each pepper cultivar which could be part of the particular features of these cultivars that are included in the European Register of protected designations of origin for these Spanish agricultural products. Besides, this provides an added value to these autochthonous products and may have some incidence at the marketing and economical levels in their respective producing sectors.

## Figures and Tables

**Table 1:** Fresh weight (FW) of whole fruits from four pepper varieties at two ripening stages.

Variety	FW Green (g)	FW Ripe Red (g)
Melchor	245.22 ± 13.41	212.05 ± 12.45
Piquillo	43.50 ± 2.62	40.91 ± 6.69
Padrón	16.19 ± 1.91	24.37 ± 1.58
Alegría	43.78 ± 1.94	36.13 ± 2.38

Data are the means ± SEM of ten fruits from each variety and ripening stage.

**Table 2:** Content of capsaicin and dihydrocapsaicin in pericarp and placenta from fruits of four pepper varieties at two ripening stages.

Variety	Ripening Stage	Tissue	Capsaicin (μg/g FW)	Dihydrocapsaicin (μg/g FW)	Capsaicin + Dihydrocapsaicin (μg/g FW)
Melchor	Green	Pericarp	0 k	0 k	0 l
		Placenta	0 k	0 k	0 l
	Red	Pericarp	0 k	0 k	0 l
		Placenta	0 k	0 k	0 l
Piquillo	Green	Pericarp	0.40 ± 0.01 i	0.56 ± 0.01 i	0.96 ± 0.02 j
		Placenta	1.35 ± 0.63 gh	0.24 ± 0.13 j	1.59 ± 0.76 hijk
	Red	Pericarp	0.25 ± 0.02 j	0.54 ± 0.01 i	0.79 ± 0.03 k
		Placenta	0.59 ± 0.03 h	0.61 ± 0.01 h	1.20 ±0.04 i
Padrón	Green	Pericarp	2.11 ± 0.08 g	0.03 ± 0.02 k	2.14 ± 0.10 gh
		Placenta	244.09 ± 34.85 c	33.10 ± 4.31 d	277.19 ± 39.16 c
	Red	Pericarp	22.45 ± 2.26 e	3.02 ± 0.19 f	25.47 ± 2.45 e
		Placenta	553.47 ± 29.59 b	166.96 ± 5.00 b	720.43 ± 34.59 b
Alegría	Green	Pericarp	8.91 ± 1.69 f	1.55 ± 0.21 g	10.46 ± 1.90 f
		Placenta	205.23 ± 9.46 c	72.96 ± 3.42 c	278.19 ± 12.88 c
	Red	Pericarp	51.06 ± 0.55 d	7.25 ± 0.35 e	58.31 ± 0.90 d
		Placenta	766.26 ± 37.00 a	269.44 ± 27.77 a	1035.70 ± 64.77 a

Placenta tissue was used once seeds were discarded. Data are the means ± SEM of three replicates determined from five fruits of the four varieties and at the two ripening stages. Different letters after each value indicate that differences were statistically significant (ANOVA, *p* < 0.05). FW, fresh weight.

**Table 3:** Total glutathione (GSH + GSSG) and the ratio GSH/GSSG from fruits of four pepper varieties at two ripening stages.

Variety	Ripening Stage	GSH + GSSG (μg·g^−1^ FW)	GSH/GSSG
Melchor	Green	88.93 ± 12.84	18.55
Melchor	Red	60.12 ± 1.38 *	32.02 *
Piquillo	Green	78.83 ± 21.43	15.04
Piquillo	Red	78.84 ± 13.10	33.71 *
Padrón	Green	65.24 ± 8.57	4.24
Padrón	Red	80.07 ± 5.75 *	5.08 *
Alegría	Green	50.08 ± 2.08	7.62
Alegría	Red	70.43 ± 4.16 *	13.89 *

GSH, reduced glutathione. GSSG, oxidized glutathione. Data are the means ± SEM of three replicates determined from five fruits of the four varieties and at the two ripening stages. Asterisks indicate significant differences of red fruits with respect to green fruits for each variety (*t*-student, *p* < 0.05). FW, fresh weight.
